# Strengths-weaknesses-opportunities-threats analysis of artificial intelligence in anesthesiology and perioperative medicine

**DOI:** 10.3389/fdgth.2024.1316931

**Published:** 2024-02-20

**Authors:** Henry J. Paiste, Ryan C. Godwin, Andrew D. Smith, Dan E. Berkowitz, Ryan L. Melvin

**Affiliations:** ^1^ Department of Anesthesiology and Perioperative Medicine, University of Pittsburgh School of Medicine, Pittsburgh, PA, United States; ^2^ Department of Anesthesiology and Perioperative Medicine, University of Alabama Birmingham School of Medicine, Birmingham, AL, United States; ^3^ Department of Radiology, University of Alabama Birmingham School of Medicine, Birmingham, AL, United States

**Keywords:** data science, artificial intelligence, SWOT, perioperative medicine, machine learning and AI

## Abstract

The use of artificial intelligence (AI) and machine learning (ML) in anesthesiology and perioperative medicine is quickly becoming a mainstay of clinical practice. Anesthesiology is a data-rich medical specialty that integrates multitudes of patient-specific information. Perioperative medicine is ripe for applications of AI and ML to facilitate data synthesis for precision medicine and predictive assessments. Examples of emergent AI models include those that assist in assessing depth and modulating control of anesthetic delivery, event and risk prediction, ultrasound guidance, pain management, and operating room logistics. AI and ML support analyzing integrated perioperative data at scale and can assess patterns to deliver optimal patient-specific care. By exploring the benefits and limitations of this technology, we provide a basis of considerations for evaluating the adoption of AI models into various anesthesiology workflows. This analysis of AI and ML in anesthesiology and perioperative medicine explores the current landscape to understand better the strengths, weaknesses, opportunities, and threats (SWOT) these tools offer.

## 1 Introduction

**Figure 1:**
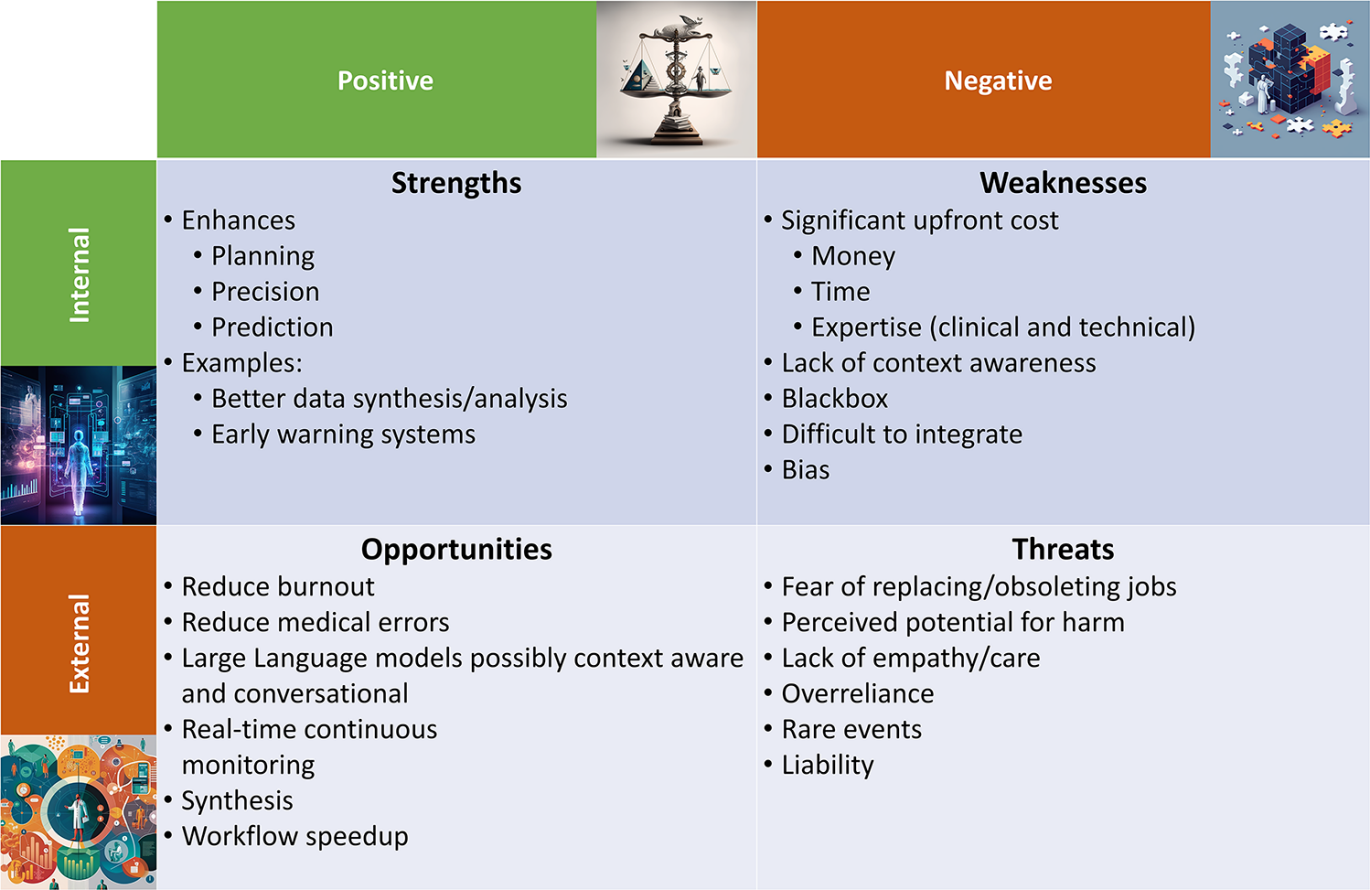
Summarized here are the strengths, weaknesses, opportunities, and threats (SWOT) of artificial intelligence (AI) in perioperative medicine. Images were generated with the Midjourney (TM, San Francisco, CA) AI using prompts suggested by OpenAI's (TM, San Francisco, CA) ChatGPT.

Artificial intelligence (AI) is rapidly emerging as a mainstay in the implementation and delivery of healthcare around the globe, and the pace of innovation, change, and growth in healthcare technologies is accelerating (1). In particular, the healthcare industry has seen significant growth in the implementation of AI over the past three decades, as the digitization of electronic medical records in the 1990s has made large-scale deployment of AI possible. Before exploring AI's strengths, weaknesses, opportunities, and threats (SWOT, Figure 1) in anesthesiology and perioperative medicine, we briefly review the current landscape of AI and ML applications in medicine.

AI applications and implementations vary widely, ranging in scope from statistical learning for risk prediction (e.g., logistic regression) to deep learning models to provide imaging diagnosis of CT scans (e.g., 3D-CNN U-Net). Some algorithms provide virtual nurses to review the vital signs of a sick patient and communicate findings with the physician (2), while other advanced imaging-based AI systems can help echocardiographers interpret challenging images (3). The adoption of AI into medical imaging has prepared other specialties for change and growth; anesthesiology and perioperative medicine are no exceptions (4, 5).

This review examines the current uses of AI today and explores what obstacles and opportunities lie ahead, as summarized in Figure 1. After a brief review of medical adaptations of AI, the remainder of the article is formatted as a SWOT assessment of AI in anesthesiology and perioperative medicine to promote discourse regarding the use of AI in anesthesiology and to establish the tools necessary for critically assessing its implementations (6).

## 2 AI in medicine

Diagnostic Radiology and Pathology are two specialties that have embraced significant adoption of AI technologies. In addition to diagnostic radiology and pathology, there have been increasing AI and ML-based technologies spanning oncology, ophthalmology, and general medicine decision making (7, 8). In terms of more simple image-based specialties, diagnostic radiology and pathology are the two clinical specialties were a most obvious entry-point for AI applications in healthcare, as they both utilize one of AI's most well-vetted skills: deep learning for computer vision (9). Applications of image classification, semantic segmentation, and object tracking have many applications outside of healthcare, and significant prior development and advanced hardware (e.g., GPUs) paved the way for quick adoption into imaging-based care. As a result, there are multitudes of readily accessible AI models for imaging and computer vision, many of which have been retrained on medical images. Outside of clinical medicine and decision making, there are emerging technologies and literature discussing and discovering AI and ML uses for medical devices (10).

Early successes of well-trained AI improved diagnostic accuracy in cancer detection. In 2017, researchers at Google used their AI to identify malignant tumors in breast cancer images and achieved an 89% accuracy rate (vs. 73% by a human pathologist).^1^ The job of a pathologist consists of much more than simply identifying a tissue pattern and noting if a sample appears benign or malignant, but the use of AI as an adjuvant supporting physician decision-making could benefit the patient and improve outcomes (in this case, by improving the turnaround time, diagnostic capability and accuracy) (11).

While pathology and diagnostic radiology may be well-suited to AI assisting their day-to-day job, how can this be deployed in anesthesiology? Imaging applications of AI have already found their way into anesthesiology practice. For example, AI-guided ultrasound already facilitates workflows in different scenarios. In many cases, ultrasound may not be necessary but can facilitate quickly and safely completing an exam. In cases of preoperative dyspnea, a bedside ultrasound may quickly rule out pneumothorax. In the case of a problematic epidural placement in labor and delivery, an ultrasound may quickly help identify spinal anatomy safely. In the case of a bedside transthoracic or transesophageal cardiac ultrasound exam, the valvular disease or arrythmia can be diagnosed quickly. AI can speed up procedures or improve diagnostic accuracy in all these cases.

AI's early-stage development and deployment into anesthesiology and perioperative medicine may utilize similar supervised learning models. Current uses of AI in anesthesiology today include predicting which patients may experience post-induction hypotension (12). In this example, the training dataset included many variables, including age, ASA status, and current medications. In this supervised learning model (i.e., the physician labeled patients with hypotension following induction for model training purposes), the ML algorithm learned that this patient suffered from hypotension. Tagged patient variables included: preoperative medications, pre-operative vital signs, medical comorbidities, induction medications, and intra-operative vital signs. ML algorithms are capable of continuous learning and, with proper monitoring and maintenance, can produce more accurate results and improved capabilities to identify combinations of variables that may place patients at the most significant risk. As these physical (e.g., ultrasound assistance) and virtual (e.g., risk-prediction) implementations of AI are emerging in the perioperative space, let us explore the SWOT of their adoption.

## 3 Strengths

There are many strengths to using AI in anesthesiology and perioperative medicine, the majority of which are categorizable into three groups *planning*, *precision*, and *prediction*. While some AI applications might fall into zero categories or more than one, the topics are broad enough to highlight the different strengths of AI in anesthesiology while providing unique forms of clinical decision support.

Preoperative planning AI tools support clinicians' awareness of potential adverse events during surgery and allows for contingency planning. While physicians factor in comorbidities and associated risks before an operation, fully integrating patient history, lab values, comorbidities, and other critical surgical factors in a detailed model is time-consuming, and it is unrealistic to manually quantify all risks for each surgical contingency or potential adverse outcome. Nevertheless, as AI tools mature and hardware advances continue, it becomes increasingly feasible to have comprehensive risk predictions for surgical trajectories and adverse events, without manual input from an anesthesiologist (13).

Precision, the ability to cater clinical care specifically to each patient's physiology, is borne from our ability to synthesize and analyze large quantities of patient-specific data rapidly. AI can integrate patient information from disparate sources to estimate previously generalized physiological parameters [e.g., specific limits of mean arterial pressure for autoregulation (14)] or to generate novel estimates of cardiovascular health [e.g., approximating arterial stiffness (15)]. Understanding an individual's detailed, real-time physiology by unlocking metrics like autoregulation limits or arterial stiffness measures can provide physicians with detailed, quantitative insights that were previously inaccessible in a traditional perioperative setting.

*Prediction* can help physicians plan for imminent emergencies and provide them with critical extra time for decision-making and action. To date, AI tools provide early warning signs of hypotension (16), sepsis (17), hypoxemia (18), unexpected intubation (19), and even mortality perioperatively (20). Integration of physiological, perioperative signals in real-time is made possible through advanced hardware, providing AI a platform to rapidly synthesize information from waveform and vital data detailing the patient's state with processing speeds that far outpace humans.

For example, in perioperative care, intraoperative hypotension and persistent desaturations on pulse oximetry may be markers of an acute pulmonary embolism. AI could detect this change in vital signs minutes before the bedside anesthetist, alerting the anesthesia care team and decreasing the time to pulmonary embolus rescue. Postoperatively, AI knows which medications and doses the patient received throughout the case and can quickly calculate a postoperative nausea and vomiting risk (21), providing the anesthesia team with detailed risk factors for clinical decision making, clinically indicated treatment, and cost savings (22). With better data synthesis and analysis, coupled with early warning systems, the triad of planning, precision, and prediction will better serve patient outcomes and the hospital bottom line.

## 4 Weaknesses

Fundamentally, AI algorithms are weighted matrices that make educated guesses that are imperfect. These algorithms can learn in two primary ways: supervised learning, or being “taught,” and unsupervised learning, or “learning from itself.” The data points these algorithmics learn from often including data created in a system with well-documented healthcare disparities (23). In particular, AI lacks context awareness, requires significant investment in hardware and personnel to create or is otherwise cost-limiting, and is vulnerable to bias and human errors in training data. This is why there must be a renewed and continued focus on encoding of existing biases. However, this again has significant up-front costs, including financial, time, personnel, and expertise. Most practicing physicians are not data scientists, computer engineers, or software developers. Therefore, developing trustworthy and ethically aligned AI algorithms requires collaboration between physicians, data scientists, and developers. There must be immense teamwork and trust between these groups and a solid foundational understanding of each other's fields to develop a medically competent and unbiased AI.

A commonly highlighted weakness of AI is that models are often shipped as a black box, where the decisions it makes are opaque to the user. Black box models provide decisions and predictions without access to model details (weights) making it unclear how these conclusions are derived. In this sense, there is a lack of “context awareness,” in regard to how a black box will operate (7). Solutions for model interpretability are available, such as saliency maps and SHAP values, which better delineate how AI functions within its boundaries. However, the extent of their utility is a topic of debate (24).

A handful of other weaknesses currently hinder the deployment of AI into more advanced settings of anesthesiology, although these issues will diminish over time as tools become increasingly sophisticated. One example is that most current AI algorithms are narrowly focused and can only execute singular, linear tasks without any clinical context or more comprehensive picture of the patient, and a significant hurdle exists in the logistical challenge of integration in a peri/postoperative setting. Any given intensive care unit will have a unique agglomeration of new and old medical equipment, and synchronizing these devices is no trivial task. The challenges of this integration and deployment, particularly in a medically complex unit like an ICU, cannot be overstated.

## 5 Opportunities

The opportunities for AI in anesthesiology are staggering. As total patient interactions and anesthetizing locations grow yearly, continual workflow improvements will help keep the anesthesiology workforce efficient and less susceptible to burnout. AI provides a tremendous opportunity to improve various aspects of the clinical workflow, effectively increasing anesthesiologist efficiency. AI-enabled workflows will reduce physician fatigue, improve the accuracy of pre-operative and perioperative diagnoses, and ultimately reduce medical errors. It is important to note that in the high-paced field of anesthesiology, AI will predominately provide supporting roles in decision-making and not function independently, at least for now. Two areas with promising opportunities include applications of generative AI like Large Language Models (LLMs) and with real-time AI for precision monitoring (25, 26).

### 5.1 Large language models

Recent development in generative AI made possible with large language models like chat-GPT are rapidly reshaping how humans work with AI (25). These tools use language-based context awareness, and some models have already passed several human exams, including the United States Medical Licensing Exam. Applications of generative AI can facilitate everything from administrative tasks to extracting unstructured data from the EHR. As Noy and Zhang showed significant time reduction (−40%) and quality improvement (+18%) for using generative AI in midlevel professional writing, we have only just begun to see how this technology can improve the work and workflow of anesthesiologists (27).

### 5.2 Real-time algorithms

Real-time algorithms can make calculations nearly instantaneously (on the order of milliseconds) and already support various aspect of closed loop control in anesthesiology (28–30). In terms of patient assessment, real-time algorithms can provide unique perioperative insights (14, 31). Whether by detecting subtle changes in a waveform indicative of an impending problem or by synthesizing across different perioperative data sources to measure something new (e.g., vascular stiffness), real-time AI for perioperative precision medicine shows promise for improving patient care and outcomes (32). The most well-known use of this today in the operating room is Bispectral Index (BIS) monitoring, which is now being combined with respiratory rate, pulse oximetry, and cardiac monitoring, with a proprietary AI algorithm, to guide anesthesiologist decision making peri-operatively in regards to depth of anesthesia.

The patient-physician relationship is particularly unique in anesthesiology. The attending physician may only have a few minutes, or less, to build rapport and trust to convey an anesthetic plan to the patient before rolling back to the operating room (33). Trying to convince a patient that physicians will use AI during their case can be a hard sell in this short window. A study showed that patients do not want AI to replace physicians, but that they are okay with a symbiosis between their physician team and clinically vetted AI applications. It will be essential to preserve and protect this patient-physician relationship while making it clear to the patient that AI will serve a peripheral but supportive role in their care. With this real time continuous monitoring, improved rate of synthesis when compared to the functional capacity of a single human brain, with a faster workflow, the opportunities to improve anesthesia care both within the operating room and outside of it are truly limitless.

## 6 Threats

There is an underlying belief among certain clinician groups that AI threatens physician job security. A recent publication indicates that medical students are concerned that diagnostic radiologists will lose their job to AI. In contrast, diagnostic radiologists are not afraid of losing their jobs but are slightly afraid of some “turf loss.” (34) These concerns are real, as AI is already replacing certain high-paying jobs. However, an anesthesiologist's ability to critically interpret the results of an AI model with a comprehensive understanding of the patient provides support for AI deployments that only operate with a human in the loop.

Two critical considerations during the development and deployment of AI, particularly in anesthesiology, are those of ethics and the preservation of the human-doctor relationship (25). Firstly, as discussed in the weaknesses, designing, building, and maintaining an ethical AI is of paramount importance. As these ML algorithms are built, it is imperative that our Hippocratic Oath remain engrained in this code. And as healthcare biases are learned by trained AI algorithms, the ethical component of AI must be kept in the forefront of our minds. To that end, it is important to consider development of kill-switches to terminate any AI applications should the Hippocratic “do no harm” oath be violated. Additionally, reporting requirements for AI failures will allow for continual assessment of model utility (35). We must continue working on developing AI in the context of the doctor-patient and an in-the-loop AI, where AI supplements patient care and augments physician knowledge and skills.

Several additional nuanced threats deserve consideration before implementing AI at scale in any healthcare network. One of these is the rare disease paradigm, in which the heavy reliance on AI to help diagnose something may misdiagnose a rare disease due to a statistical unlikelihood, further delaying the time to correct diagnosis. From an anesthesiology perspective, this is less probable. However, this is still a concern given that the AI may “miss” a pulmonary embolus or something rarer and view it as idiopathic hypoxemia, for example (36). So long AI and ML continue to serve in the capacity as an adjunct and additive to patient care, these risks are small and are heavily outweighed by the downsides. It will remain paramount to not become over reliant on this technology and remember, even during critical components of the case, that the technology itself cannot see the patient, but only the data points of the patient.

## 7 Discussion

AI is currently in its infancy within the specialty of anesthesiology, as demonstrated by the relatively small number of approved algorithms compared to, say, Radiology (7). This review presents several different schools of thought regarding where AI can go in anesthesiology. One school of thought is to deploy AI liberally across as many settings as possible (OR, PACU, clinics, ICUs) and integrate speedily, as the potential benefits of AI in these settings are innumerable. On the other hand, one thought is to approach AI deployment cautiously, deploying one algorithm into one setting and monitoring closely for mistakes and opportunities to correct, in other words, to “play it safe.”

Additionally, there are differing opinions on where in anesthesiology AI can first be deployed. In many ways, the ICU is a hospital floor where anesthesia-adjacent medical problems evolve over hours to days, vs. the hyperacuity of the operating room, where situations unfold over seconds to minutes. However, AI implementations have initially been done in small, isolated situations for specific research projects, predominantly as proof of concept (37). Utilizing retrospective turned prospective studies, we can deploy AI models in safe yet valuable ways to verify and validate their utility in the perioperative setting. Ultimately, more comprehensive AI applications will likely see deployments in full clinical settings, such as the ICU or operative settings, but this future remains years to decades away.

In the anesthesia and perioperative setting, many possibilities exist for a similar AI-based intervention as seen in imagining fields, that is, to help identify mistakes (38). Regarding quality improvement, AI can prompt warnings of missing values (39) and recommend a lab study for a patient in pre-op (40) that may help guide perioperative anesthetic care. In clinical decision-making, AI can interpret mean arterial pressures and help guide controlled profusion regulation based on a patient-by-patient basis of circulatory autoregulation limits, for example (14).

The potential future developments of AI in anesthesiology and perioperative medicine are the most exciting parts to consider (38). Imagine a hospital or health network where patients are preoperatively optimized in their surgeon's clinic, and the physician anesthesiologist is notified the next time they log in to their electronic medical record, “Mr. Smith is undergoing non-urgent hernia mesh repair tomorrow with an 8% chance of a code event. Click to see the full report.” Then, during the case, a score is available in the operating room and on the operating room board of other risk factors or percent-chance of adverse outcomes in this patient, updating in real-time. While we remain years to decades away from well-implemented, system-wide deployment of AI across a health system (38), we continue to see incremental improvements and opportunities to utilize AI in anesthesiology. As a conceptual example, consider having AI read pulse oximetry waveform data in real-time and alerting physicians seconds to minutes before impending hypoxemic respiratory failure. This advanced alerting system would give the anesthesiologist extra time to prepare for intervention. These small steps will continue to propel AI forward in a data-driven future of anesthesiology.

## 8 Conclusion

It is an incredibly exciting time to be in medicine and specifically to be on the cutting-edge of technological advances, like AI, in anesthesiology and perioperative medicine. While AI remains in its infancy in medicine, specialties including radiology, pathology, and others, have had significant strides in improving patient outcomes and physician satisfaction with their implementation of AI. As summarized in Figure 1, the future of medicine will most certainly utilize AI in some way, although it remains unclear to what extent. AI will likely supplement, augment, or otherwise assist physician decision-making in the future, and we must remember that to err is absolutely human (41).

## Footnotes

^1^Liu Y, Gadepalli K, Norouzi M, et al. Detecting Cancer Metastases on Gigapixel Pathology Images. Published online 2017:1–13. http://arxiv.org/abs/1703.02442
